# Supplementary material for: Nonenzymatic assembly of active chimeric ribozymes from aminoacylated RNA oligonucleotides
Source: Proc Natl Acad Sci U S A. 2022 Feb 9;119(7):e2116840119. doi: 10.1073/pnas.2116840119 (PMC8851484; doi:10.1073/pnas.2116840119)
Supplement: Supplementary File [file pnas.2116840119.sapp.pdf]

PNAS
www.pnas.org

Supplementary Information for

Nonenzymatic assembly of active chimeric ribozymes from aminoacylated RNA oligonucleotides

Aleksandar Radakovic[a,b,c,d,1], Saurja DasGupta[a,b,c,d,1], Tom H. Wright[a,b,c,d], Harry R.M. Aitken[a,b,c,d], Jack W. Szostak[a,b,c,d,e*]

[a]HHMI, Massachusetts General Hospital, Boston, MA 02114;

[b]Department of Molecular Biology, Massachusetts General Hospital, Boston, MA 02114;

[c]Center for Computational and Integrative Biology, Massachusetts General Hospital, Boston, MA 02114;

[d]Department of Genetics, Harvard Medical School, Boston, MA 02115; and

[e]Department of Chemistry and Chemical Biology, Harvard University, Cambridge, MA 02138

[1]These authors contributed equally

*To whom correspondence should be addressed: Jack W. Szostak, email: szostak@molbio.mgh.harvard.edu

This PDF file includes:

Figures S1 to S12

Table S1

Supplementary Figures

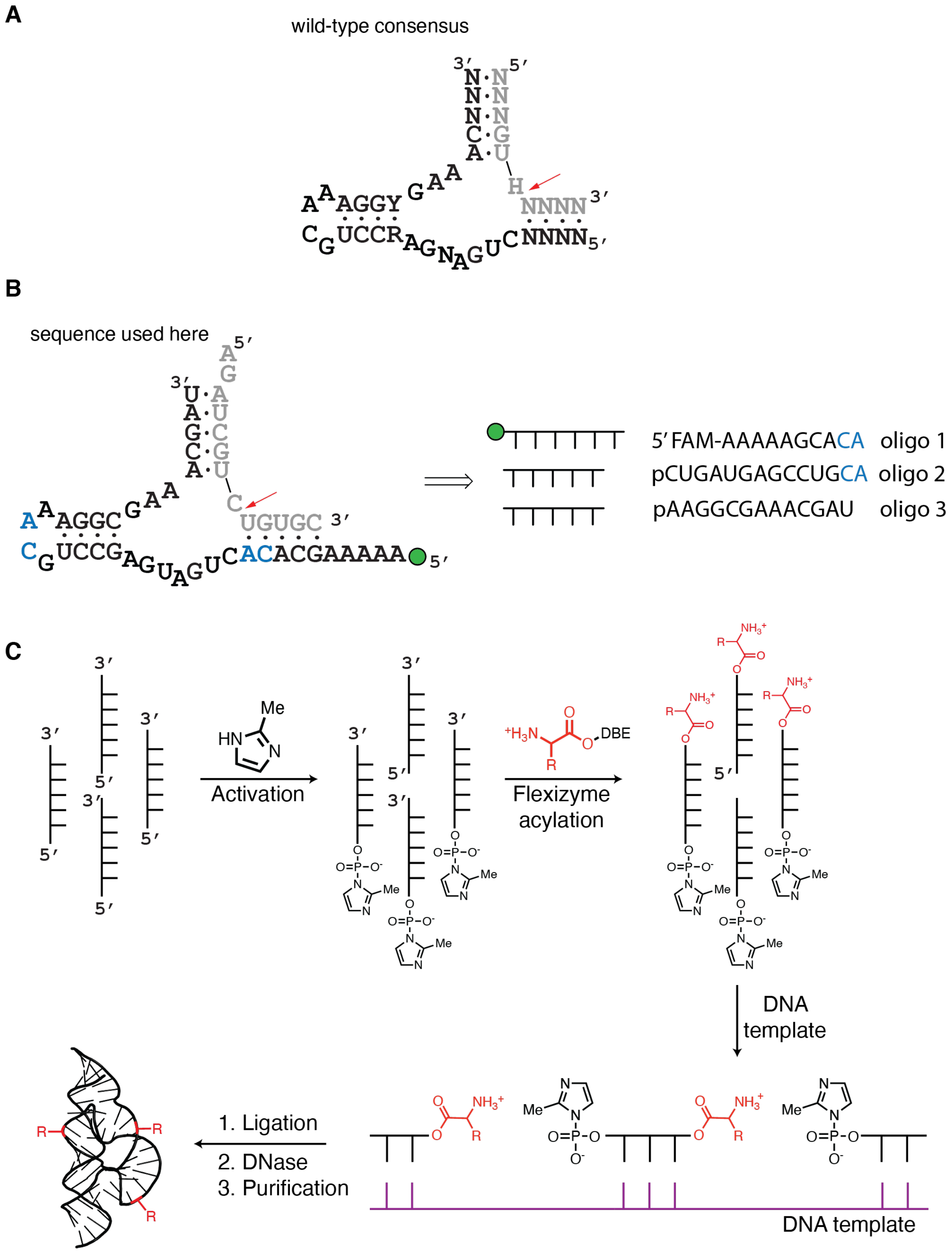

Figure S1. Hammerhead mutation and deconstruction. A The wild-type consensus sequence of the hammerhead ribozyme. **B** Sequence variant used in this work to accommodate flexizyme-catalyzed aminoacylation. The hammerhead variant sequence was assembled from three oligonucleotides of roughly equal length (designated oligos 1-3). Blue nucleotides represent the 5′-CA-3′ dinucleotides that are the required substrates for the flexizyme-catalyzed aminoacylation. 5′ A_5 sequence was added to increase aminoacylation yield. Red arrow represents the cleavage site in the RNA substrate. The green circle represents the FAM label. The “p” prefix represents the 5′ phosphate. **C** Diagram of the method used to assemble chimeric ribozymes. Oligonucleotides that comprise the ribozymes are first activated with 2-methylimidazole. The oligonucleotides, except for the 3′ terminal oligonucleotide, are then aminoacylated using the flexizyme ribozyme and 3,5-dinitrobenzyl esters of amino acids. Following the addition of a DNA template, ligation occurs, and subsequent digestion of the template with DNase allows isolation of the ribozyme by preparative urea-PAGE.

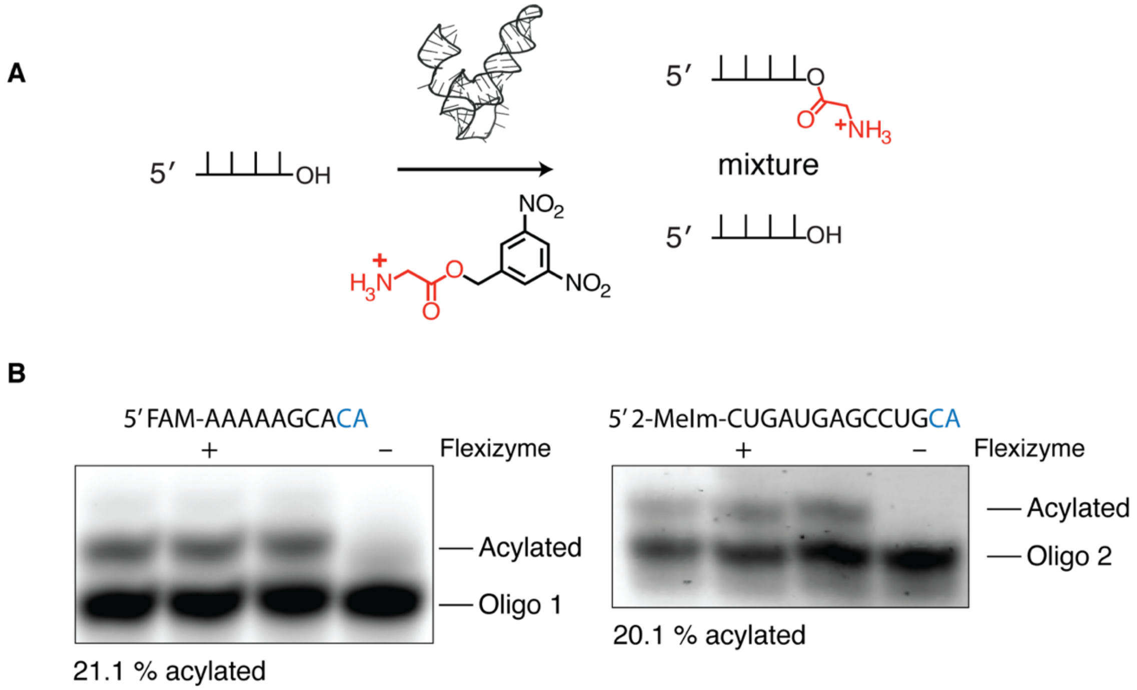

Figure S2. Aminoacylation of RNA oligonucleotides that comprise the hammerhead ribozyme. A Schematic of the flexizyme-catalyzed aminoacylation reaction. flexizyme accepts 3,5-dinitrobenzyl esters of amino acids as substrates and specifically aminoacylates the 3′ OH of the 2′,3′ diol of RNA oligonucleotides. The reaction does not proceed to completion and thus yields a mixture of aminoacylated and non-aminoacylated RNA. **B** Aminoacylation of oligos 1 and 2 at 0 ºC was monitored by 20 % denaturing acid urea-PAGE (0.1 M NaOAc pH 5.0, 7 M urea; running buffer 0.1 M sodium acetate pH 5.0). The aminoacylated oligonucleotide migrates slower than the non-aminoacylated oligonucleotide and full resolution of the two bands allows quantification of the aminoacylation yield. Oligo 1 was fluorescently labeled, thereby allowing direct quantification, whereas oligo 2 was stained with SYBR Gold for quantification. The yields below each gel represent an average of triplicate measurements.

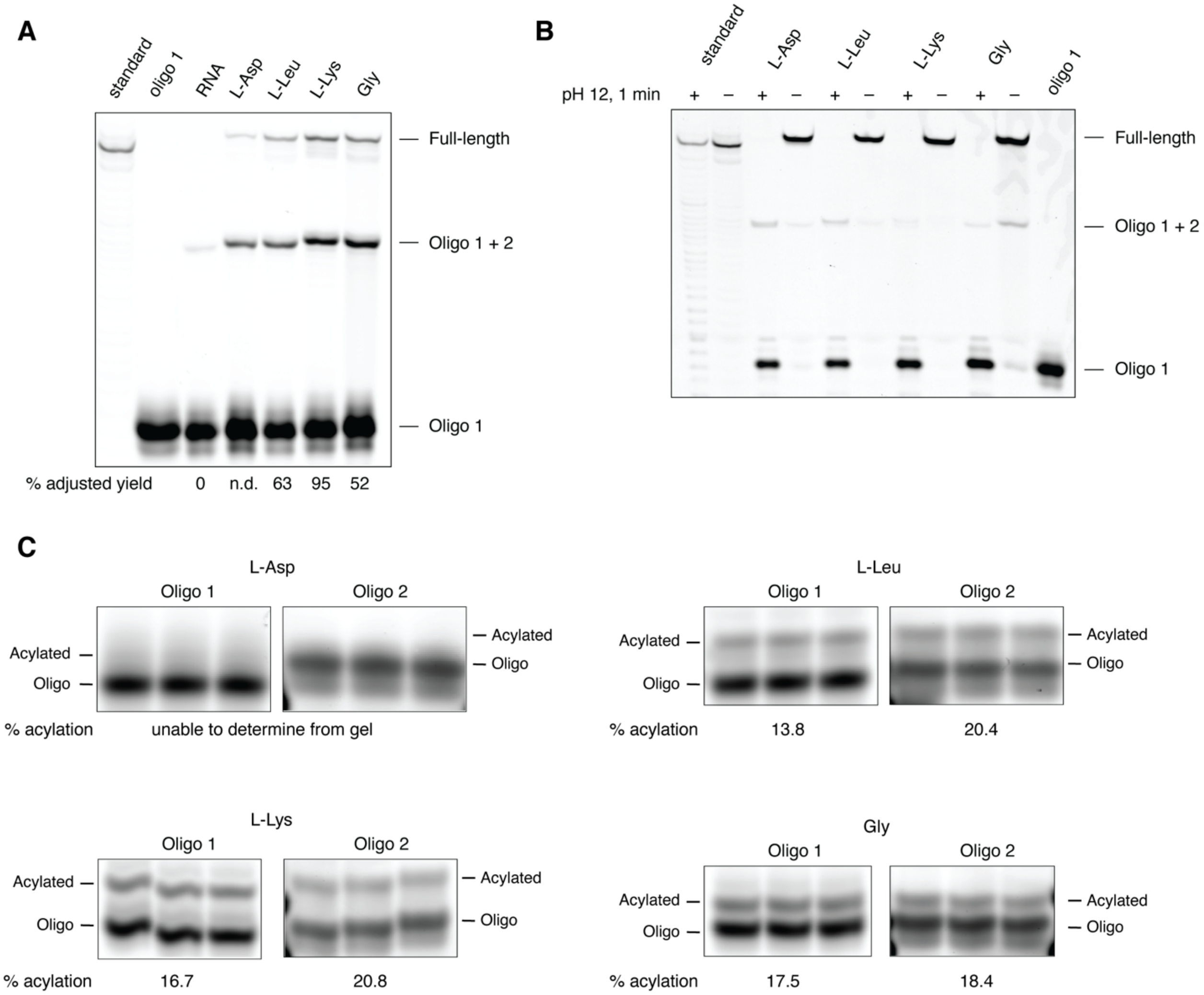

Figure S3. Chimeric hammerhead assembly with four different amino acids. A A representative denaturing 20 % urea-PAGE gel of the hammerhead assembly reactions. The true yield of the L-Asp assembly reaction could not be determined due to the poor resolution of the aminoacylated and non-aminoacylated bands by acidic urea-PAGE. The standard was a 5′ FAM-labeled hammerhead ribozyme sequence purchased from IDT. **B** Each purified, chimeric ribozyme was subjected to transient alkaline conditions by the addition of 200 mM NaOH for 1 minute. After the NaOH treatment, all four chimeric ribozymes were hydrolyzed such that no full-length product was detectable. The RNA standard displayed minor non-specific hydrolysis. A faint band that corresponds to “Oligo 1 + 2” is still visible due to the incomplete hydrolysis and/or background RNA reaction during assembly. **C** Acidic urea-PAGE analysis of aminoacylation reactions for the four different amino acids. Percent acylation represents the average of technical triplicates. These values were used to normalize the assembly yield as described in Methods. L-Asp aminoacylated RNA could not be resolved from non-aminoacylated RNA, hence the adjusted yield for this assembly could not be calculated.

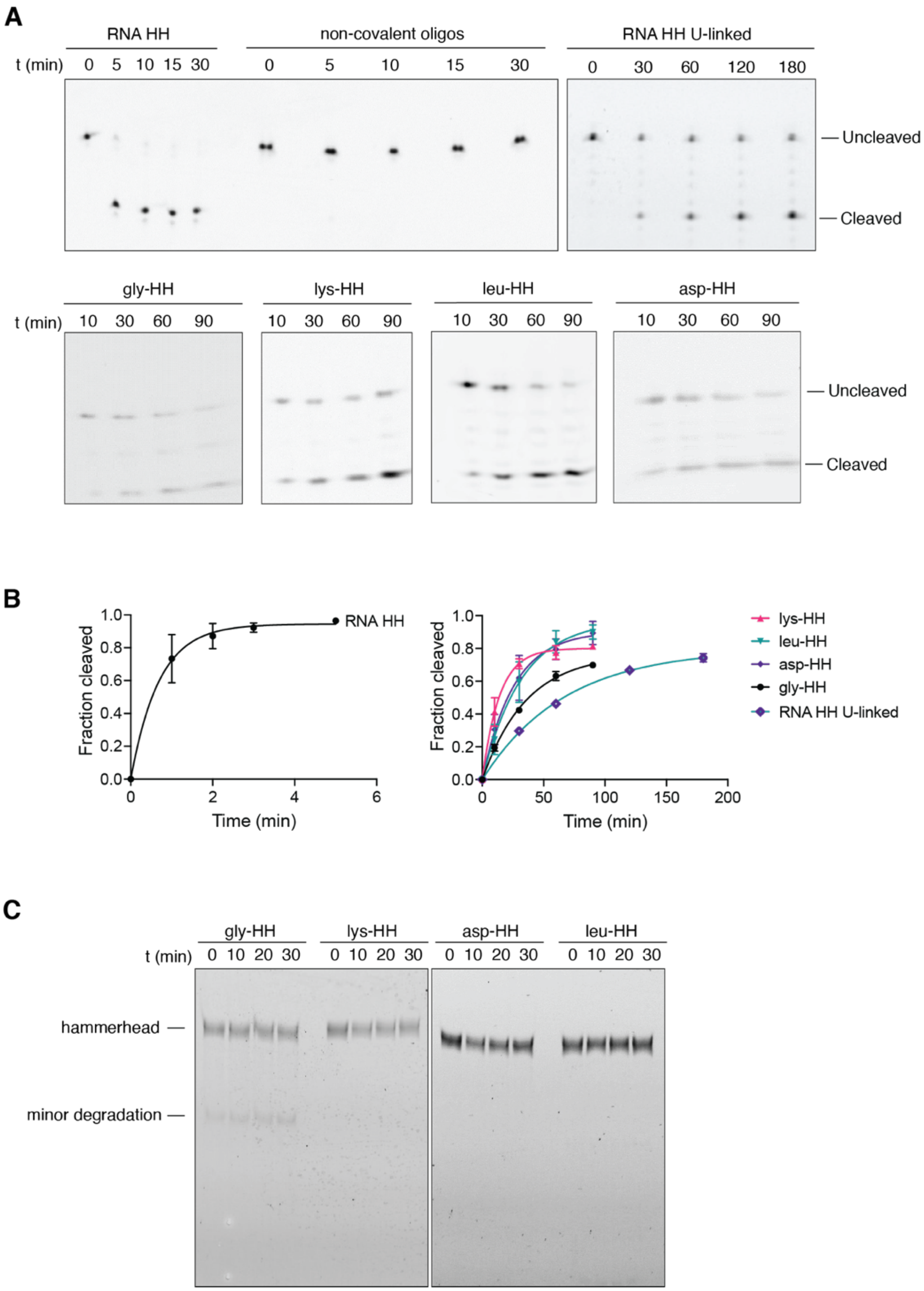

Figure S4. Hammerhead cleavage kinetics and degradation. A Representative denaturing 20 % urea-PAGE gels of cleavage reactions for different hammerhead constructs. RNA HH is the all-RNA hammerhead; non-covalent oligos are the three hammerhead oligos without any covalent linkages; RNA HH U-linked is the all-RNA hammerhead with single U nucleotides in place of each amino acid bridge. **B** Kinetic plots of the hammerhead cleavage reactions. Left: all-RNA hammerhead cleavage. Right: chimeric and U-linked hammerhead cleavage. **C** Representative

denaturing 20 % urea-PAGE gels of chimeric hammerhead ribozymes incubated at reaction conditions without the addition of the substrate.

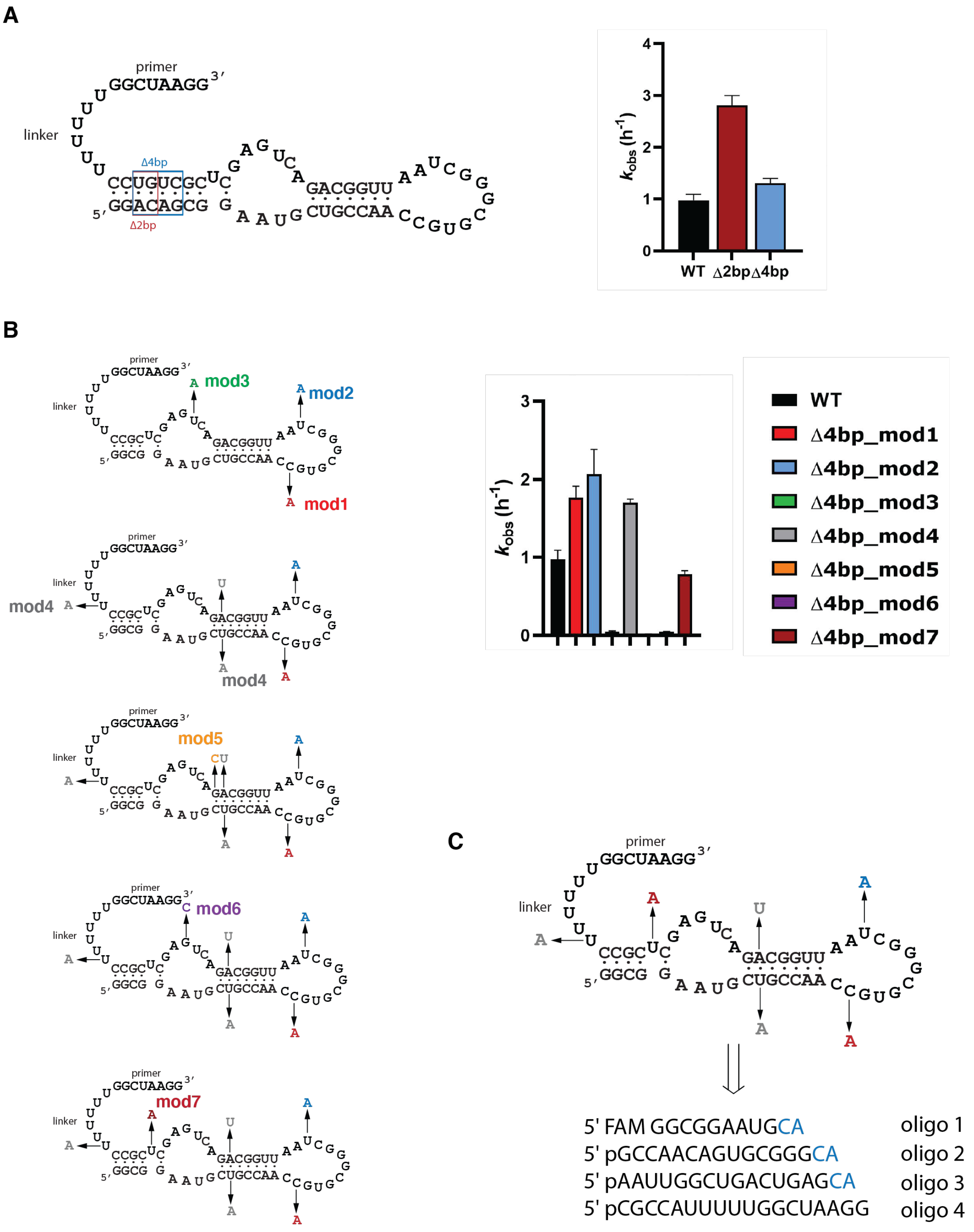

Figure S5. Optimization of 2AI ligase ribozyme sequence. A Truncations of the base pairs within the blue and red rectangles yielded shorter ribozymes that retained the catalytic RNA ligation activity. **B** The 4 bp deletion mutant from **A** was altered to install the 5′-CA-3′ dinucleotide required for flexizyme substrates. The sequence labeled “mod7” displayed comparable catalytic RNA ligation activity to the 4 bp deletion mutant and was selected for the assembly experiments. **C** The

“mod7” ligase ribozyme was assembled from four oligonucleotides ranging from 11 nt to 18 nt in length (designated oligos 1-4). Sequences labeled in blue represent the aminoacylation sites. The “p” prefix represents the 5′ phosphate.

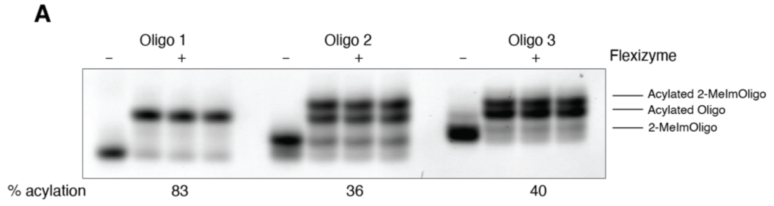

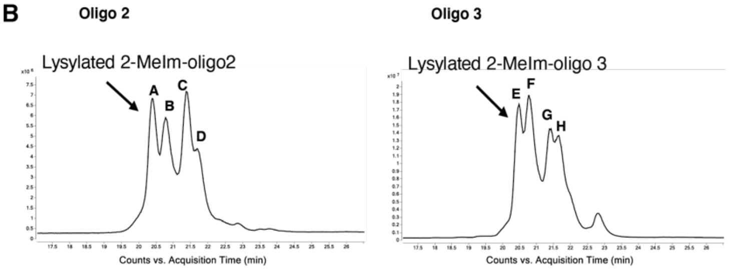

Signal	Calculated	Found	Identity
A	5427.8745	5427.7797	Lysylated 2-MeIm-oligo 2
B	5363.8320	5363.7192	Lysylated oligo 2
C	5299.7795	5299.6727	2-MeIm-oligo 2
D	5235.7370	5235.6360	oligo 2
E	5719.8729	5719.7686	Lysylated 2-MeIm-oligo 3
F	5655.8304	5655.7277	Lysylated oligo 3
G	5591.7779	5591.6872	2-MeIm-oligo 3
H	5527.7354	5527.6432	oligo 3

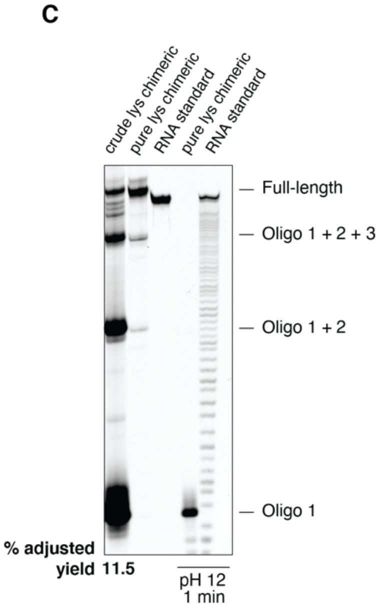

Figure S6. Assembly of the chimeric RNA ligase from lysylated oligonucleotides. A Acidic urea-PAGE analysis of aminoacylation reactions for the three different oligonucleotides stained with SYBR Gold. The lysylated oligonucleotide migrates more slowly than the non-lysylated oligonucleotide. Additional bands present in oligo 2 and oligo 3 samples represent lysylated unactivated oligonucleotides due to hydrolysis of 2-methylimidazole during acidic gel purification. **B** Total ion chromatograms for oligos 2 and 3 that were used to determine the identity of the additional bands shown in **A**. **C** A representative denaturing 20 % urea-PAGE gel of the L-lys ligase assembly reaction. Assembly reaction was performed as described in the Methods, and the adjusted yield was the average of technical triplicates. The standard was a 5′ FAM-labeled ligase sequence purchased from IDT. The purified chimeric ribozyme was subjected to transient alkaline conditions by the addition of 200 mM NaOH for 1 minute. After the NaOH treatment, the chimeric ribozyme was hydrolyzed such that no full-length product was detectable. The RNA standard displayed minor non-specific hydrolysis.

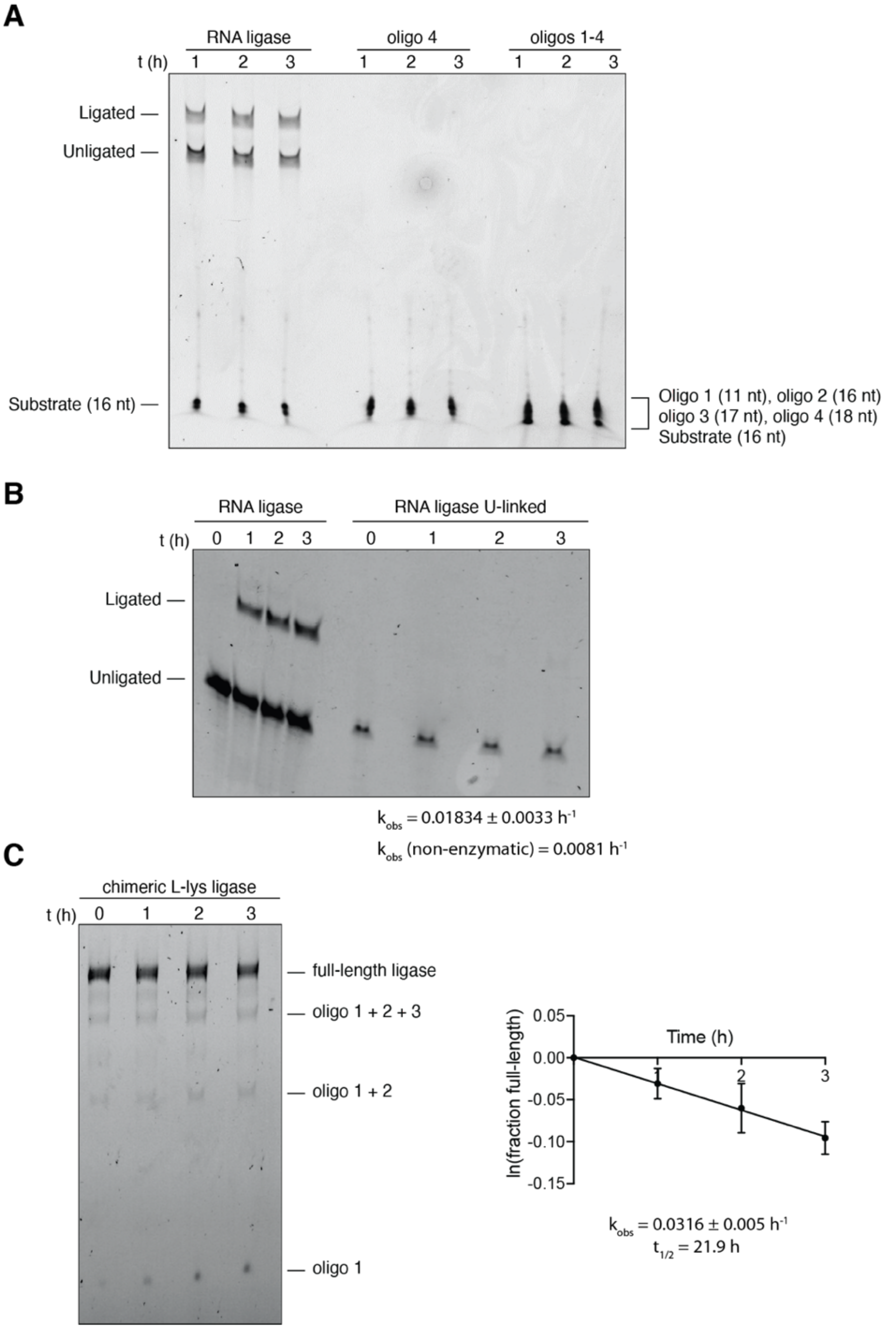

Figure S7. Non-covalent and U-linked RNA ligase activity and chimeric L-lys ligase degradation. A Representative denaturing 10 % urea-PAGE gel of RNA ligase activities. RNA ligase is the all-RNA ligase ribozyme serving as the control for enzymatic ligation. Oligo 4 is the 3′ oligonucleotide fragment of the ligase (see Figure S5C) that contains the nucleophile in the ligation reaction, and it was used as the control for any nonenzymatic ligation between itself and the substrate. Oligos 1-4 are the four non-covalently linked fragments of the ligase incubated with the substrate. **B** Representative denaturing 10 % urea-PAGE gel showing the lack of activity of the all-RNA ligase with single U nucleotides in place of each amino acid bridge. **C** Left: representative denaturing 10 % urea-PAGE gel showing the degradation of the chimeric L-lys RNA ligase under the ligation conditions without any substrate present (see Figure S5C for the identity of oligos 1, 2,

and 3). Right: kinetic plot of the degradation reaction. Half-life was calculated using the formula $\ln(2)/k_{obs}$.

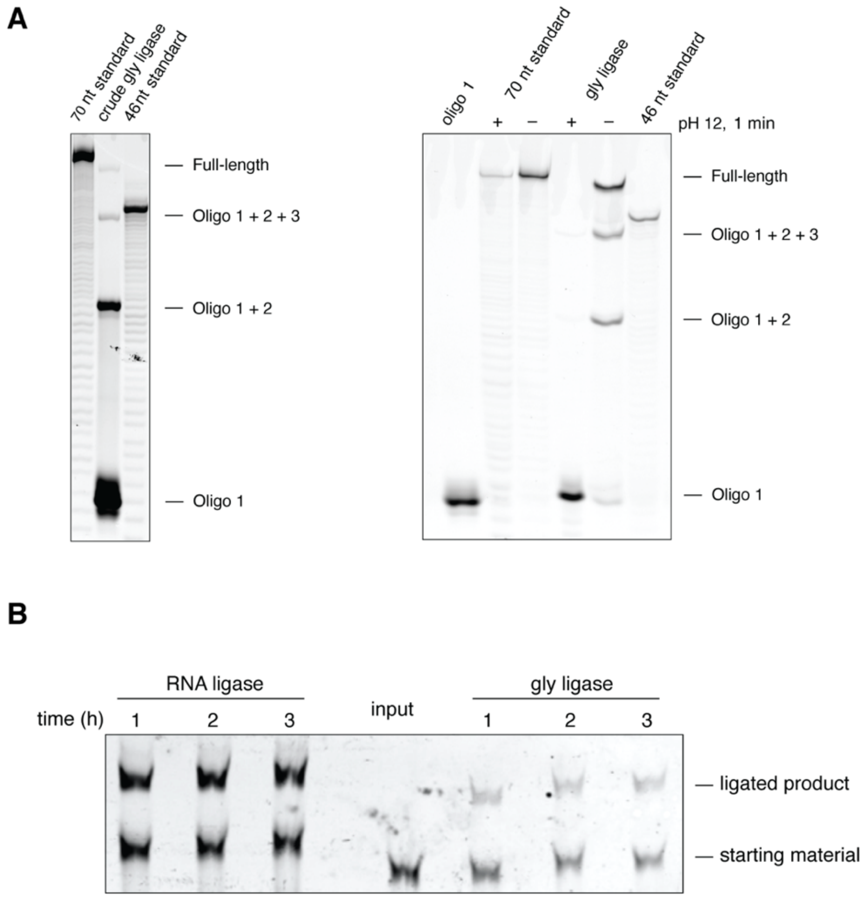

Figure S8. Assembly of a chimeric RNA ligase from glycylated oligonucleotides. A Left: A representative denaturing 20 % urea-PAGE gel of the gly ligase assembly reaction. Assembly reaction was performed as described in the Methods. The adjusted yield was not calculated. The two standards used were 5′ FAM-labeled 70 nt and 46 nt sequences purchased from IDT. Right: The purified chimeric ribozyme was subjected to transient alkaline conditions by the addition of 200 mM NaOH for 1 minute. After the NaOH treatment, the chimeric ribozyme was hydrolyzed such that no full-length product was detectable. The RNA standards displayed minor non-specific hydrolysis. **B** A representative denaturing 10 % urea-PAGE of the gly-ligase ligation reaction over time. The ligation product band is clearly visible; however, due to low ligation yields caused by competing hydrolysis of the aminoacyl ester linkages in the product and ribozyme, we did not determine the product yields.

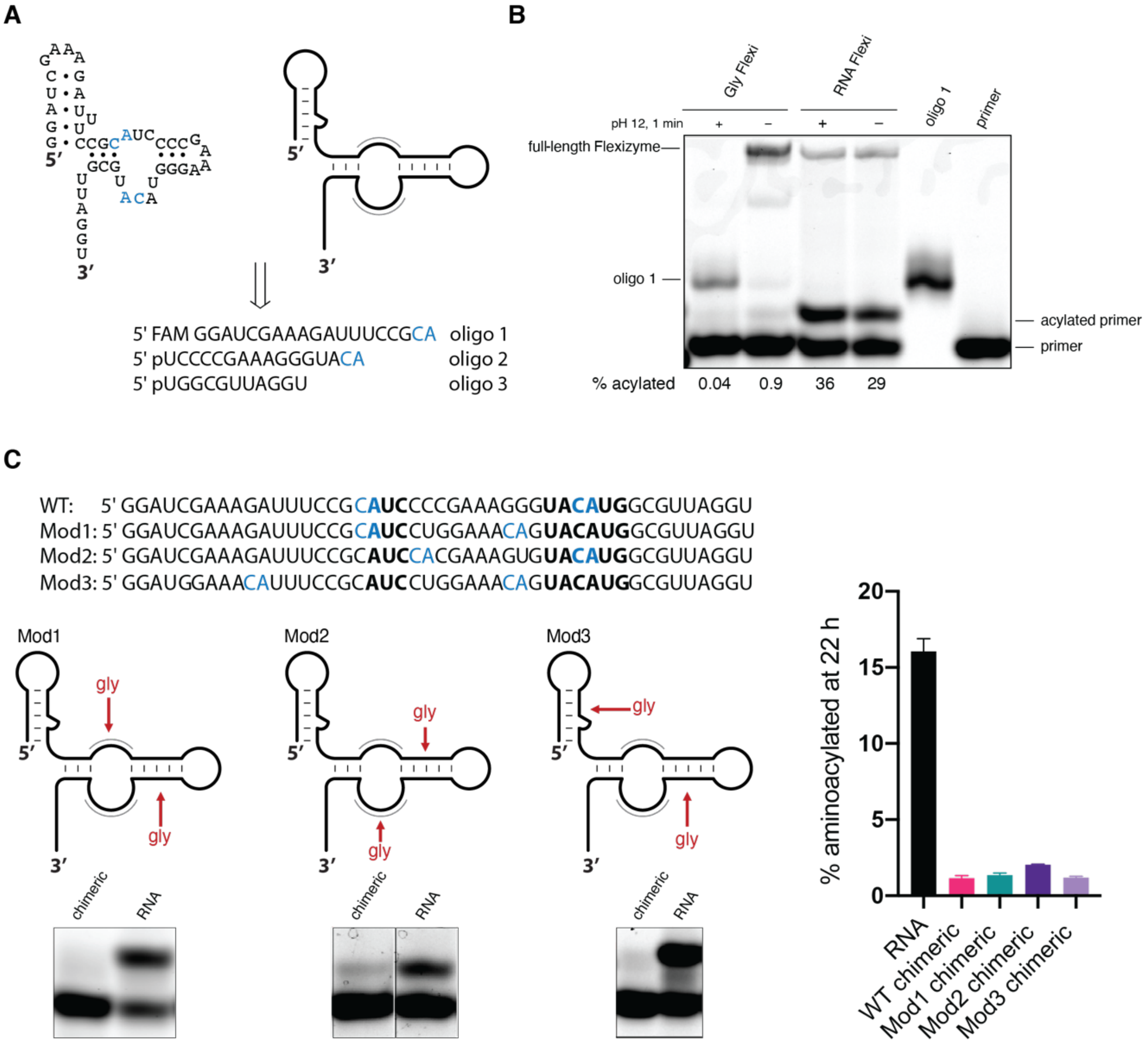

Figure S9. A functional chimeric flexizyme can be assembled from glycylated RNA. A Left: The wild-type flexizyme sequence; right: diagram of flexizyme used hereafter. CA sequences that serve as substrates for the flexizyme aminoacylation are shown in blue. The flexizyme was assembled from three oligonucleotides (designated oligos 1-3). The “p” prefix represents the 5′ phosphate. **B** A representative denaturing 20 % acidic urea-PAGE used to monitor the flexizyme activity at the 22 hour time point. The gly chimeric flexizyme retained the aminoacylating activity of its all-RNA counterpart, which disappeared after transient alkaline treatment. **C** The wild-type flexizyme sequence was mutated to reposition the CA sequences and gly bridges outside the catalytic center, which is indicated by a grey outline. Each flexizyme variant was modestly active as seen from representative denaturing 20 % acidic urea-PAGE. Aminoacylation yields of each ribozyme were determined by averaging triplicate measurements (right).

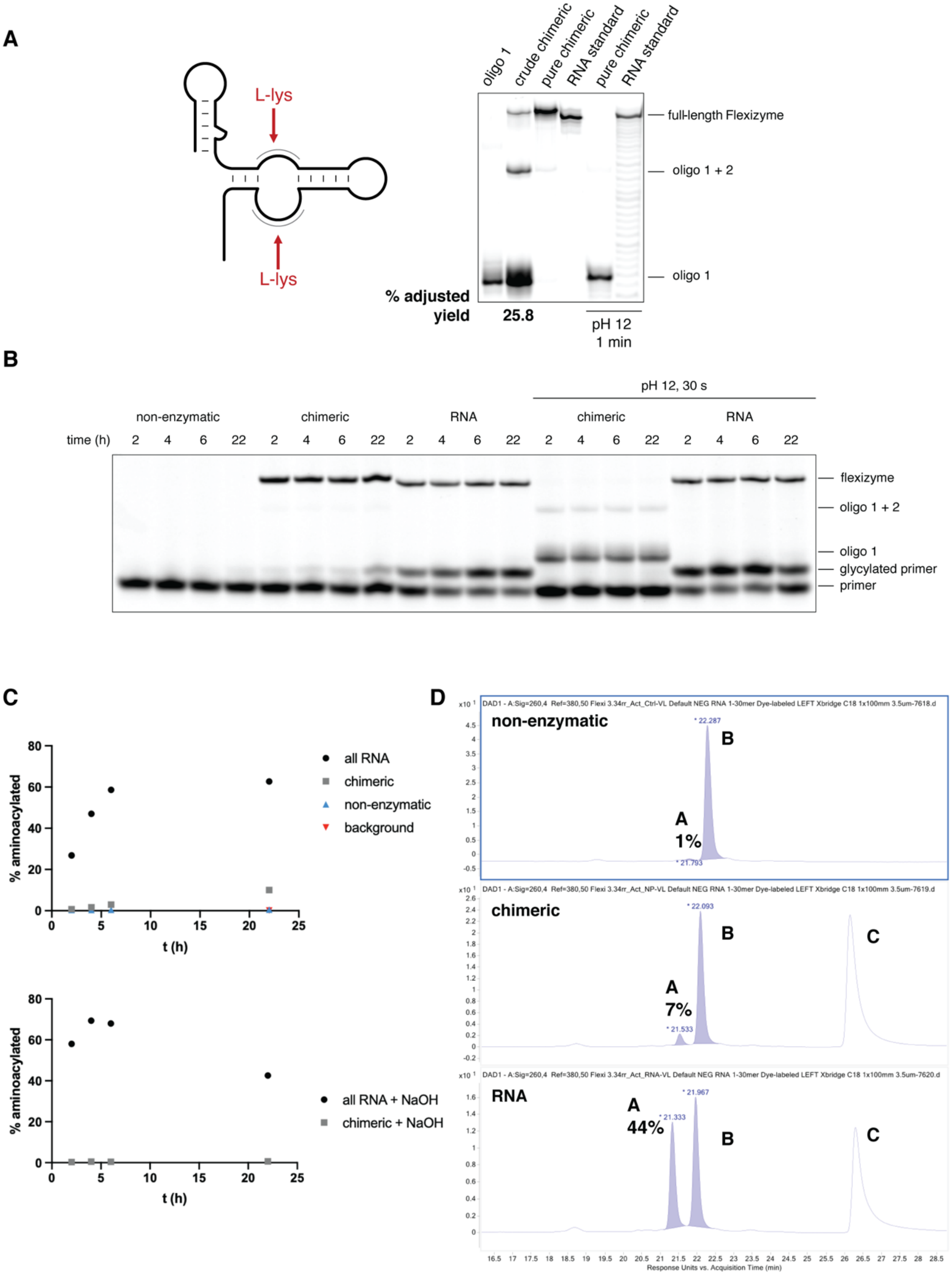

Signal	Calculated	Found	Identity
A	3822.6720	3822.6312	glycylated primer
B	3765.6506	3765.6234	primer
C	N/A	N/A	flexizyme

Figure S10. Chimeric flexizyme assembled from L-lysylated RNA is functional. A Left: diagram of the flexizyme with the L-lys “bridges” in the catalytic center indicated by the red arrows. Right: representative denaturing 20 % urea-PAGE of the L-lys flexizyme assembly reaction. The purified chimeric ribozyme was subjected to transient alkaline conditions by the addition of 200 mM NaOH for 1 minute. After the NaOH treatment, the chimeric ribozyme was hydrolyzed such that no full-length product was detectable. The RNA standard displayed minor non-specific hydrolysis. **B** A representative denaturing 20 % acidic urea-PAGE used to monitor the flexizyme activity over time. The nonenzymatic reaction included all components except for the flexizyme. The chimeric L-lys flexizyme aminoacylates the primer substrate with glycine only if it is not pretreated with 200 mM NaOH. The RNA standard flexizyme retains its activity even after the alkaline pretreatment. **C** Top: the aminoacylation activity was monitored over time in triplicate and the average percent aminoacylation was plotted. The background value was determined by loading the pure primer substrate and quantifying the amount of signal at the gel location parallel to the aminoacylated band. Bottom: aminoacylation activity monitored over time after the pretreatment with 200 mM NaOH for 30 seconds. **D** UV chromatograms of the three reactions collected at the 22 hour time point of the aminoacylation reaction. Integrating the signals that corresponded to the aminoacylated primer and primer resulted in values that roughly match the gel analysis. Some hydrolysis of the aminoacylated primer is expected during LC-MS analysis, hence the lower values compared to the gel. The calculated and observed m/z for each UV signal are tabulated, confirming that aminoacylation with glycine occurs.

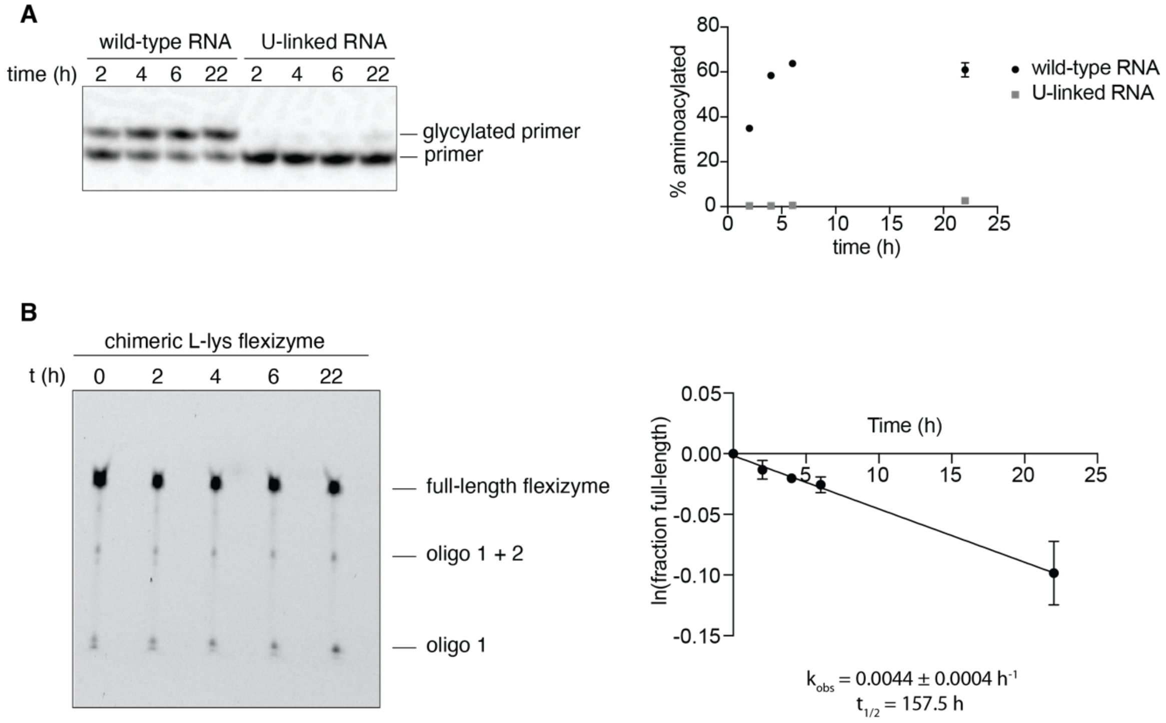

Figure S11. U-linked RNA flexizyme activity and chimeric L-lys flexizyme degradation. A Left: Representative denaturing 20 % acidic urea-PAGE gel of flexizyme activities; wild-type RNA is the all-RNA flexizyme ribozyme serving as the control for aminoacylation; U-linked RNA is the all-RNA flexizyme ribozyme that contains single U nucleotides in place of each amino acid bridge. Right: kinetic plot of the aminoacylation reaction over time performed in triplicates. **B** Left: representative denaturing 20 % urea-PAGE gel showing the degradation of the chimeric L-lys flexizyme under the aminoacylation conditions without any substrate present (see Figure S9A for the identity of oligos 1 and 2). Right: kinetic plot of the degradation reaction. Half-life was calculated using the formula $\ln(2)/k_{obs}$.

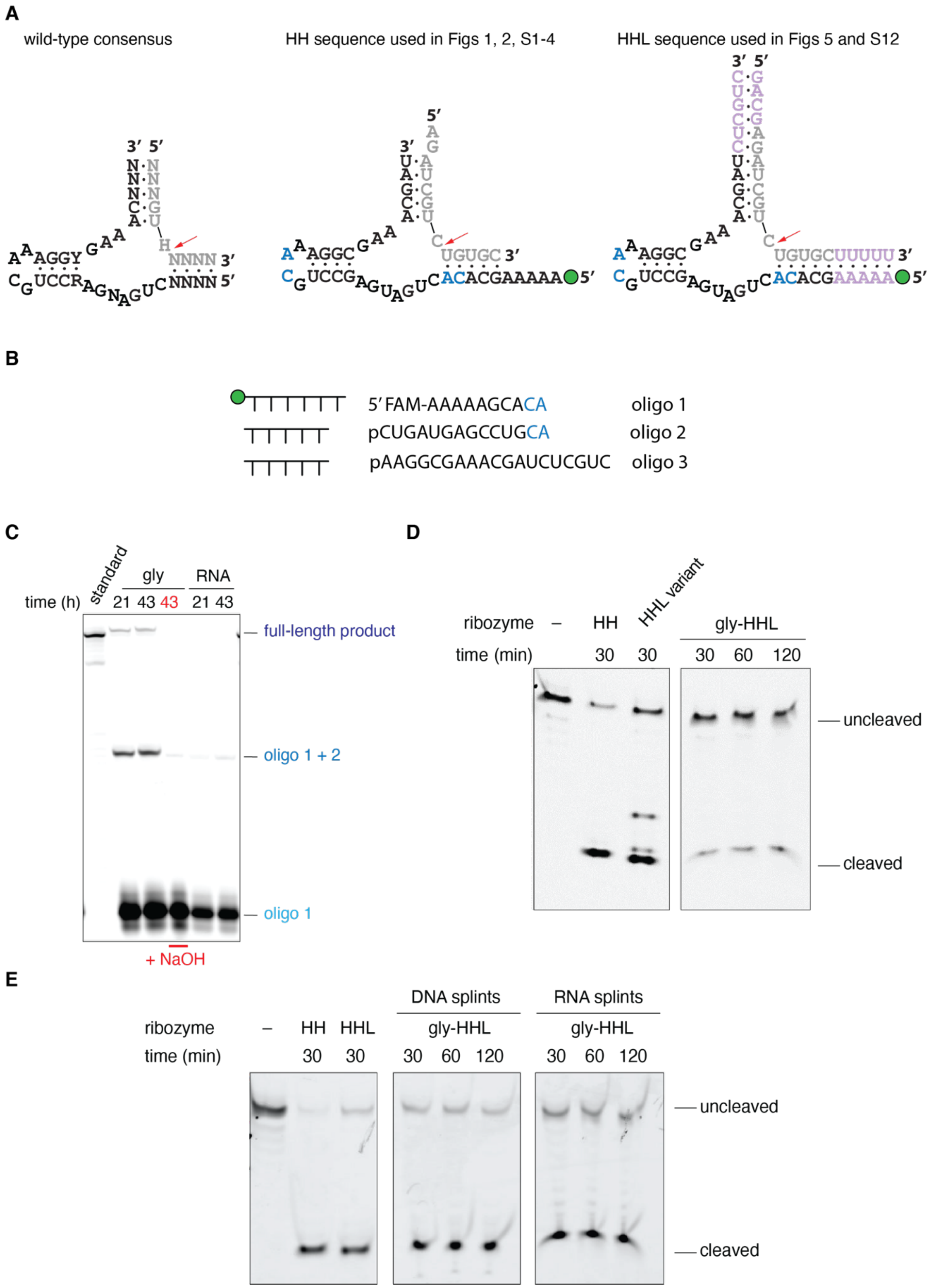
A
wild-type consensus
HH sequence used in Figs 1, 2, S1-4
HHL sequence used in Figs 5 and S12
B
5′ FAM-AAAAAGCACA oligo 1
pCUGAUGAGCCUGCA oligo 2
pAAGGCGAAACGAUCUCGUC oligo 3
C
standard
gly
RNA
time (h) 21 43 43 21 43
full-length product
oligo 1 + 2
oligo 1
+ NaOH
D
ribozyme – HH HHL variant gly-HHL
time (min) 30 30 30 60 120
uncleaved
cleaved
E
DNA splints
RNA splints
ribozyme – HH HHL gly-HHL gly-HHL
time (min) 30 30 30 60 120 30 60 120
uncleaved
cleaved

Figure S12. Chimeric hammerhead assembled using splints from glycylated oligonucleotides cleaves its substrate in the same pot. A Diagram of the hammerhead ribozymes used in this study. Red arrow indicates the cleavage site. **B** Oligonucleotides used in the splint-assisted assembly of the chimeric HHL ribozyme. **C** Urea-PAGE of an RNA splint-assisted assembly reaction. Treatment of the glycylated assembly reaction with 200 mM NaOH for 1 minute resulted in the disappearance of the full-length product band, indicating that the product was gly-bridged. **D** One-pot hammerhead cleavage reaction at 25 ºC. Lanes HH and HHL show substrate cleavage by the all-RNA control ribozymes. Lanes labeled gly-HHL show time-dependent one-pot substrate cleavage by the chimeric HHL ribozyme assembled on RNA splints. **E** One-pot hammerhead cleavage reaction at 42 ºC.

Supplementary Table

Table S1: Sequences used in this work. Nucleotides in purple are deoxyribonucleotides, while all others are ribonucleotides. 2-MeImp represents 2-methylimidazole activated phosphate. 2-AImp represents 2-aminoimidazole activated phosphate.

Name	Sequence	Use
dFx flexizyme M1	5′-GGAUCGAAAGAUUUCCGCAUCCCC GAAAGGGUACAUGGCGUUAGCU	Figures 1-5, S2,3,6,8-10,12
dFx flexizyme M2	5′-GGAUCGAAAGAUUUCCGCAUCCCC GAAAGGGUACAUGGCGUUAGUU	Figures 1-5, S2,3,6,8-10,12
HH oligo 1	5′-FAM-AAAAAGCACA	Figures 1, 2, S1-4
HH oligo 2	5′-2-MeImpCUGAUGAGCCUGCA	Figures 1, 2, S1-4
HH oligo 3	5′-2-MeImpAAGGCGAAACGAU	Figures 1, 2, S1-4
HH oligo 3L	5′-2-MeImpAAGGCGAAACGAUCUCGUC	Figures 5, S12
HH RNA template	5′-AUCGUUUCGCCUUUGCAGGCUCAUCAGUGUGCUUUUU	Figure 1
HH DNA template	5′-ATCGTTTCGCCTTTGCAGGCTCATCAGTGTGCTTTTT	Figures 2, S3
HH standard	5′-FAM-AAA AAG CAC ACU GAU GAG CCU GCA AAG GCG AAA CGA U	Figures 1,2, S3
HHL standard	5′-FAM-AAA AAG CAC ACU GAU GAG CCU GCA AAG GCG AAA CGA UCU CGU C	Figures 5, S12
HH substrate	5′-FAM-AGAUCGUCUGUGC	Figures 2, S4
HH DNA splint 1	5′-ATCAGTGTGC	Figures 5, S12
HH DNA splint 2	5′-GCCTTTGCAG	Figures 5, S12
HH RNA splint 1	5′-AUUGGUGUGU	Figure S12
HH RNA splint 2	5′-GUUUUUGUAG	Figure S12
HHL substrate	5′-Cy5-GACGAGAUCGUCUGUGCUUUUU	Figures 5, S12
Ligase oligo 1	5′-FAM-GGCGGAAUGCA	Figures 3, S5-8

Ligase oligo 2	5′-2-MeImpGCCAACAGUGCGGGCA	Figures 3, S5-8
Ligase oligo 3	5′-2-MeImpAAUUGGCUGACUGAGCA	Figures 3, S5-8
Ligase oligo 4	5′-2-MeImpCGCCAUUUUUGGCUAAGG	Figures 3, S5-8
Ligase DNA-RNA template	5′-CCTTAGCCAAAAATGGCGUGCTCAGTCAGCCAAUUU GCCCGCACTGTTGGCUGCATTCCGCC	Figures 3, S6,8
Ligase standard	5′-FAM-GGCGGAAUGCAGCCAACAGUGCGGGCAAAUUGGCUGACUGAGCACGCCAUUUUUGGCUAAGG	Figures 3, S6,7
70-nt standard	5′-FAM-GGACAGCGGAAUGCUGCCAACCGUGCGGGCUAAUUG GCAGACUGAGCUCGCUGUCCUUUUUUGGCUAAGG	Figure S8
46-nt standard	5′-FAM-GGAUCGAAAGAUUUCCGCAUCCCC GAAAGGGUACAUGGCGUUAGCU	Figure S8
Ligase substrate	5′-2-AimpACCACCGCAUUCCGCA	Figures 3, S7,8
Ligation Template	5′-GCGGUGGUCCUUAGCC	Figure 3, S7,8
dFx Flexizyme	5′-GGAUCGAAAGAUUUCCGCAUCCCC GAAAGGGUACAUGGCGUUAGGU	Figure S9
Flexizyme oligo 1	5′-FAM-GGAUCGAAAGAUUUCCGCA	Figures 4, S9-11
Flexizyme oligo 2	5′-2-MeImpUCCCCGAAAGGGUACA	Figures 4, S9-11
Flexizyme oligo 3	5′-2-MeImpUGGCGUUAGGU	Figures 4, S9-11
Flexizyme DNA template	5′-ACCTAACGCCATGTACCCTTTCGGGGATGCGGAAAT CTTTCGATCC	Figures 4, S9,10

Flexizyme standard	5′-FAM-GGAUCGAAAGAUUUCCGCAUCCCCGAAAGGGUACA<br>UGGCGUUAGGU	Figures 4, S9,10
Flexizyme mod1 oligo 1	5′-FAM-GGAUCGAAAGAUUUCCGCA	Figure S9
Flexizyme mod1 oligo 2	5′-2-MeImpUCCUGGAAACA	Figure S9
Flexizyme mod1 oligo 3	5′-2-MeImpGUACAUGGCGUUAGGU	Figure S9
Flexizyme mod1 DNA template	5′-ACCTAACGCCATGTACTGTTTCCAGGATGCGGAAAT CTTTCGATCC	Figure S9
Flexizyme mod1 standard	5′-FAM-GGAUCGAAAGAUUUCCGCAUCCUGGAAACAGUACA<br>UGGCGUUAGGU	Figure S9
Flexizyme mod2 oligo 1	5′-FAM-GGAUCGAAAGAUUUCCGCAUCCA	Figure S9
Flexizyme mod2 oligo 2	5′-2-MeImpCGAAAGUGUACA	Figure S9
Flexizyme mod2 oligo 3	5′-2-MeImpUGGCGUUAGGU	Figure S9
Flexizyme mod2 DNA template	5′-ACCTAACGCCATGTACACTTTCGTGGATGCGGAAAT CTTTCGATCC	Figure S9
Flexizyme mod2 standard	5′-FAM-GGAUCGAAAGAUUUCCGCAUCCACGAAAGUGUACA<br>UGGCGUUAGGU	Figure S9
Flexizyme mod3 oligo 1	5′-FAM-GGAUGGAAACA	Figure S9
Flexizyme mod3 oligo 2	5′-2-MeImpUUUCCGCAUCCUGGAAACA	Figure S9
Flexizyme mod3 oligo 3	5′-2-MeImpGUACAUGGCGUUAGGU	Figure S9

Flexizyme mod3 DNA template	5′-ACCTAACGCCATGTACTGTTTCCAGGATGCGGAAAT GTTTCCATCC	Figure S9
Flexizyme mod3 standard	5′-FAM-GGAUGGAAACAUUUCCGCAUCCUGGAAACA<br><br>GUACAUGGCGUUAGGU	Figure S9
Flexizyme substrate	5′-FAM-AGAGAAGCCA	Figures 4, S9-11
